# Magnetic bead-based adsorption strategy for exosome isolation

**DOI:** 10.3389/fbioe.2022.942077

**Published:** 2022-08-16

**Authors:** Sun Jiawei, Chen Zhi, Tian Kewei, Li Xiaoping

**Affiliations:** ^1^ Shulan International Medical College, Zhejiang Shuren College, Hangzhou, China; ^2^ Zhejiang University School of Medicine, Hangzhou, China

**Keywords:** exosomes, extracellular vesicles, exosome isolation, magnetic bead, ultracentrifugation

## Abstract

Exosomes, one type of extracellular vesicle (EV) secreted by cells, participate in intercellular communication and other biological processes as carriers of lipids, functional proteins, mRNAs, miRNAs, lncRNAs, and DNA fragments. Their presence in biofluids makes them attractive candidates as innovative clinical diagnostic tools. However, the conventional isolation and analysis of high-purity exosomes in clinical application is challenging, with traditional methods facing a number of shortcomings, including low yield or purity, long periods of processing, high cost, and difficulties in standardization. In this study, we provide an overview of commonly used exosome isolation approaches with a focus on magnetic bead-based capture, an ideal methodology with high purity and integrality of exosomes. The current challenges on exosome isolation methods are also described to highlight areas for future research and development.

## Introduction

Currently, molecular testing in biopsy samples has become a committed step in diagnosis, prognosis, and individualized therapy of disease in the era of precision medicine. At present, the tumor sample of a patient was obtained by surgery or puncture. However, tissue biopsy cannot always be performed because of the invasiveness of surgery and puncture. Moreover, results from a single biopsy might not provide sufficient real-time information to characterize the genetic heterogeneity of disease (Gerlinger et al., 2012; Swanton, 2012). Compared with the tissue biopsy, liquid biopsy (including circulating tumor cells (CTCs), circulating tumor DNA (ctDNA), and exosomes) is based on the non-invasive collection measure, convenient storage of samples, and fast acquisition of information at different stages of disease progression (Herrero et al., 2019).

**FIGURE 1:**
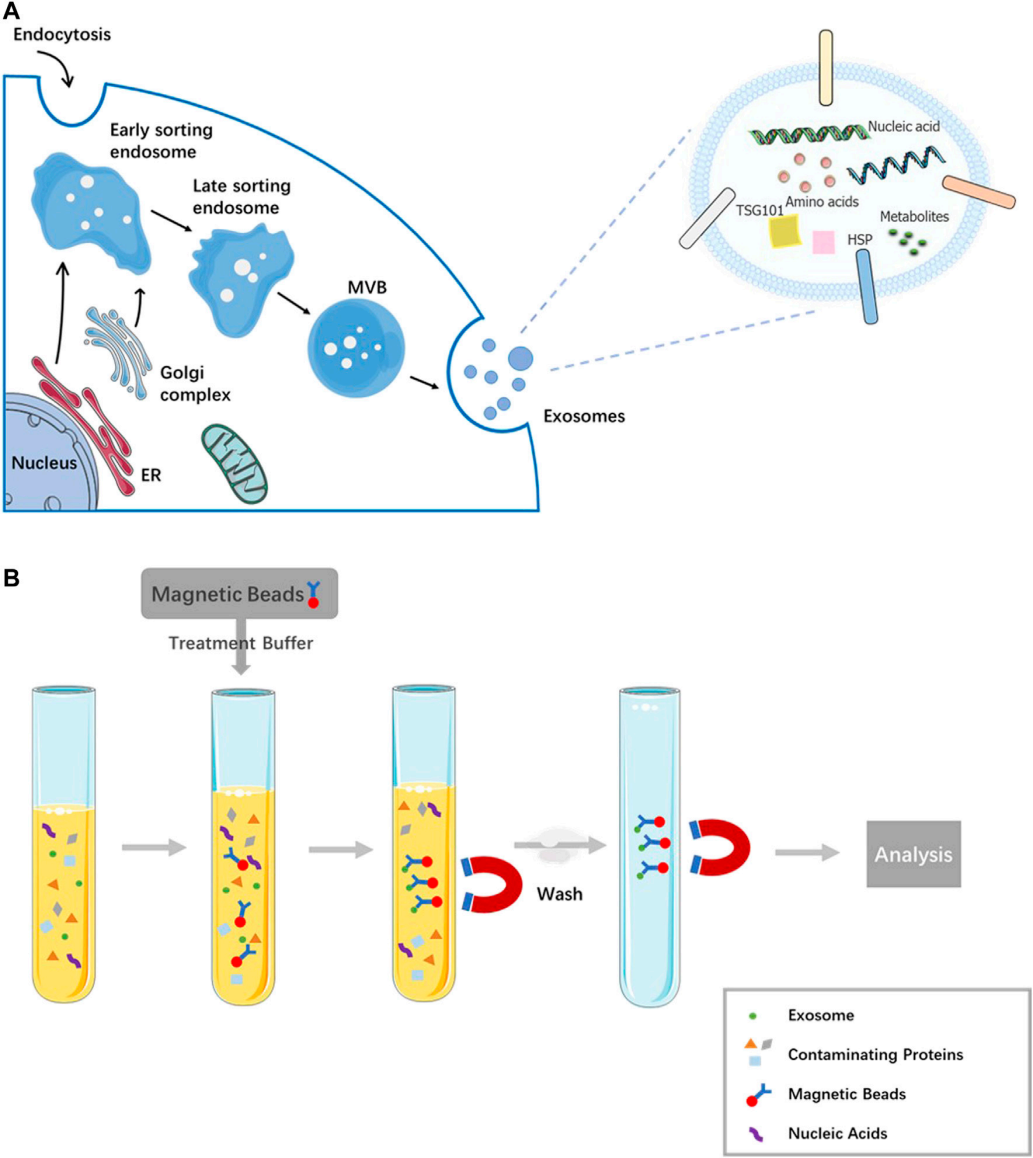
Biogenesis and its contents of exosome and magnetic bead affinity exosome isolation method. **(A)** The process production and overall composition of an exosome. **(B)** The scheme of magnetic bead affinity for exosome extraction.

Exosomes (Figure 1A), one type of extracellular vesicle (EV) secreted by cells, with size ranging from 30 to 150 nm in diameter (Raposo and Stoorvogel, 2013; Pegtel and Gould, 2019), participate in the intercellular communication and other biological processes as carriers of lipids, functional proteins, mRNAs, miRNAs, lncRNAs, and DNA fragments (Rani et al., 2011; Colombo et al., 2014; Wortzel et al., 2019). In various biological fluids including plasma, lymph, urine, saliva, ascites, saliva, and bronchoalveolar lavage fluid, exosomes can be found. At present, exosomes were widespread detected diagnostic biomarkers owing to their vital roles as monitor in different stages of disease progression (Zhang et al., 2015; Kalluri, 2016; Li et al., 2019).

Exosomes are detectable in various biofluids, but the detection and analysis of exosomes are interfered because a large numbers of biomacromolecules are present in these biofluids (Yang et al., 2020; Zhu et al., 2020). The complexity of biological samples and the heterogeneity of exosomes increased the difficulty of extraction and separation of exosomes, exploring the method of exosome isolation, and enrichment from complex biofluids for clinical detection is efficiently urgent. The main exosome isolation methods include ultracentrifugation, ultrafiltration, immunomagnetic isolation, and microfluidics (Yang et al., 2017; Sidhom et al., 2020). Among them, the most widely used approach is ultracentrifugation (UC), but it has deficiencies such as poor effectiveness for viscous liquids, requirement for expensive equipment, time-consuming, and ineffectiveness of distinguishing between exosome subsets or other particles of similar size and density. The mechanism of immunomagnetic isolation protocols is using magnetic beads coated with anti-marker antibodies to capture exosomes by recognizing the specific signature receptors on their surface. This method has advantages such as low primary sample volume, high accuracy, and no chemical contamination. The aim of the review is to summarize the roles of magnetic beads in exosome isolation.

## Exosomes isolation techniques

In recent years, the separation and enrichment technology of exosomes in body fluid samples has developed rapidly, and various innovative technologies and new platforms are emerging, which play a key role in further exploring exosomes. At the same time, because the contents of biological samples are complex and changeable, it is still technically difficult to separate exosomes efficiently. Here we summarize the principle of different isolation methods and discuss their advantages and disadvantages (Table 1).

**TABLE1:** Comparison of the current exosome extraction methods.

Method	Principle	Advantages	Disadvantages	Yield	Purity	Time	Equipment/material cost, $	Reference
Ultracentrifugation	Centrifugation and ultracentrifugation steps	Cost-effective	Time consuming	Low	High	2–20h	∼3000/10	Johnstone et al. (1989); Cvjetkovic et al. (2014); Gudbergsson et al. (2016); Helwa et al. (2017)
			Large primary sample size					
		Suitable for large volume preparation	Low accuracy					
			Contamination with media proteins					
			Time consuming					
Ultrafiltration	Centrifugation and filtration	Large primary sample size	Low portability	Low	High	∼20h	1000/20	Cheruvanky et al. (2007); Lobb et al. (2015); Konoshenko et al. (2018)
			Sensitive to centrifugation time					
Immunoaffinity enrichment	Nano-magnetic bead	Low primary sample volume	Costly	High	High	∼1h	0/650	Tauro et al. (2012); Greening et al. (2015)
		High accuracy						
Microfluidics	Microfluidic devices	Low primary sample volume	High-price	High	High	<2h	4217/1400	Chen et al. (2010); Zhang et al. (2016)
		Easily automated and integrated with diagnosis						

Ultracentrifugation, the gold standard exosome isolation method, is the most commonly used method to extract exosomes from cell biological fluid and culture supernatant (Gudbergsson et al., 2016; Helwa et al., 2017). Exosomes can be separated using size differences in the ultracentrifugation approach (Johnstone et al., 1989). Although ultracentrifugation is considered as the gold standard for exosome separation, it is tedious and time-consuming to separate exosomes by ultracentrifugation; in addition, high impurities including lipoprotein and protein, structure corruption of exosomes, and the high price of the device discourage the adoption of ultracentrifuge in the exosome separation. By this method, a number of factors, such as rotor type, ultracentrifugation time, and liquid viscosity influence the purity and yield of exosomes (Cvjetkovic et al., 2014).

In the protocol of ultrafiltration, a membrane with a specified pore size is used to separate a predetermined range of particles (Cheruvanky et al., 2007; Lobb et al., 2015; Konoshenko et al., 2018). This protocol can be used as a stand-alone isolation technique; meanwhile, it can also play the role as a complement to ultracentrifugation. After exosomes be separated from proteins *via* ultracentrifugation, membranes are used to sieve cells and large EVs. Ultrafiltration can cause the pores of the membrane to be blocked by vesicles, thus shortening the service life of the membrane and reducing the separation efficiency (Li et al., 2017). Some exosomes can also be attached to the membranes, which interfere downstream analysis, resulting in a decrease in yield and even false-positive or false-negative detection results.

The immunoaffinity capture technology has strong specificity to screen and separate exosomes selectively. Generally, the EVs with CD9, CD63, CD81, and other proteins on the membrane surface are considered as exosomes (Tauro et al., 2012). Because only a subset of exosomes expressing antibody recognition protein is captured, the yield is usually insufficient, but its purity is much higher than that of exosomes separated according to the physical properties of exosomes (Greening et al., 2015). With the progression of tumor, the specific antibodies may lose their recognition ability; in addition, the surface antigen may also be blocked or shielded, resulting in the antigen–antibody unable to combine normally, so the target exosomes cannot be obtained.

The microfluidic method, which can be applicable to exosome separation and downstream analysis, is a promising development direction of liquid biopsy in the future for its high efficiency and easy operability. Exosomes are targeted by the binding with specific antibodies immobilized on the inner capture surface of microfluidic devices (Chen et al., 2010; Zhang et al., 2016). However, the application of microfluidic technology in the exosome separation is still immature, and the yield and purity are insufficient (Contreras-Naranjo et al., 2017). Therefore, many people pay attention to synergistically apply other technologies including immunomagnetic beads with microfluidic technology for the exosome biopsy (Sharma et al., 2018).

## Magnetic bead-based exosome isolation

Specific capture of exosomes, which is closely related to immunoaffinity, is suitable for isolating by targeting specific markers on the membrane of exosome. Antibody-coated beads can be used to enrich by targeting exosome membrane markers, such as CD9, CD63, ALIX, and the epithelial cell adhesion molecule (EpCAM) (Greening et al., 2015). Latex beads have been used in the passive absorption of purified exosomes. The protocol is cheap, easy to recover from the solution while involving numerous centrifugation steps, and is challenging in terms of reproducibility (Théry et al., 2001). An alternative to latex beads for exosome capture, magnetic bead-based technology (Figure 1B), is attributed to the differences in specific interactions between receptors and ligands and other physical characteristics of exosomes (Jara-Acevedo et al., 2019).

The isolation of exosomes by magnetic beads includes two steps generally. First, exosomes from biofluid or pre-enriched by ultracentrifugation are captured by magnetic beads utilizing immunoaffinity, and then intact exosomes can be released from beads in the buffer. To enrich EVs and EV-associated miRNA efficiently, a two-step magnetic bead-based (2MBB) method is proposed for the isolation of exosomes as well as associated miRNAs from samples. Exosome-associated miRNAs are extracted by a second set of magnetic beads coated with complementary oligonucleotides after the enrichment of EV using magnetic beads. The result of RT-PCR demonstrated high efficiency of 2MBB in the EV enrichment (74 ± 7%, *n* = 4) and miRNA isolation (91 ± 4%, *n* = 4) (Chen et al., 2020). Several studies found a sandwich-type immunocomplex can be constructed for specific isolation and accurate quantification of exosomes. After exosomes specifically being captured by immunomagnetic beads, different types of nanoprobes are fixed on the surface of exosomes by hydrophobic interactions between cholesterol and lipid membranes, thus forming a sandwich-type immunocomplex. The immunocomplex can be magnetically captured and produce enhanced detectable signals (He et al., 2017; He et al., 2018; Huang et al., 2018; Tian et al., 2018; Zeng et al., 2021).

Tumor-specific exosomes are small in number, isolating total of them is complex to achieve, while the immunoaffinity bead-based method has been confirmed to be able to capture several types of tumor-specific exosomes. A 10-uL aliquot of magnetic beads coated with an anti-CD34 antibody, which is a unique marker of acute myeloid leukemia (AML), can isolate all the AML-specific exosomes from 100 to 1,000 μl AML plasma (Hong et al., 2014). The anti-epithelial cell adhesion molecule (EpCAM) tagged bead scan can be used to obtain highly pure circulating tumor-derived exosomes of ovarian cancer and esophageal squamous cell carcinoma patients (Taylor and Gercel-Taylor, 2008; Zhao et al., 2019). A CSPG4-coated magnetic bead can capture CSPG4+ exosomes produced by melanoma cells. The efficiency of immune-based capture of melanoma-derived exosomes obtained from the plasma of melanoma patients is around 95% (Sharma et al., 2018). To obtain prostate cancer-related exosomes, immunomagnetic beads coated with an anti-prostate-specific membrane antigen (PSMA) antibody can be applied to isolate them from the plasma of prostate cancer patients (Mizutani et al., 2014).

The immunomagnetic method is executed by different antibodies coated on the magnetic beads to target the surface markers of exosomes. A direct exosome isolation strategy using anti-human CD81 antibody-coated magnetic beads is able to enrich exosomes from T lymphocyte cell culture without the pre-enrichment step (Pedersen et al., 2013). Tim4, a phosphatidylserine receptor, can recognize the phosphatidylserine on the surface of EVs. Wataru N. et al. (Nakai et al., 2016) developed a practical and effective method using magnetic beads bound with Tim4 to purify exosomes and adding Ca^2+^ chelators to release exosomes from Tim4 easily. Monoclonal anti-HLA DP, DQ, and DR antibodies can be coated on magnetic beads to target exosomes derived by antigen-presenting cells (Clayton et al., 2001).The CD63-1 aptamer/magnetic bead complex formed by incubating can isolate exosomes from the colon and breast cancer cell culture supernatant effectively (Song et al., 2020). Combining the traditional immunomagnetic bead-based protocol and the microfluidic method results in benefits from both the high purity of the former and the automated continuous superiority (Niu et al., 2020).

Furthermore, some scholars found strategies of isolating exosomes in a generic way using physical or other properties of exosomes by magnetic beads. We know that the EV surface contains phosphatidylserine with negative charge. ExoCAS-2, a magnetic bead-based ion exchange platform for isolating exosomes attempts to separate exosomes by polycationic polymer-coated magnetic beads from plasma. The yield provided by ExoCAS-2 is 6.6-fold higher than UC. High purity and batch-to-batch repeatability are also unique features of ExoCAS-2 (Kim and Shin, 2021). A biofunctionalized magnetic bead with high affinity Ti(IV) ions and the insertion of a phospholipid derivative, 1,2-distearoyl-sn-glycero-3-phosphoethanolamine, is shown to effectively isolate exosomes with low contamination, a high recovery rate (>80%), and a short separation time (<1h) from the urine of prostate cancer patient (Sun et al., 2021).

Multiple studies have compared immunomagnetic beads and other current exosome isolation methods in terms of yield, purity, and operation difficulty. Comparation of common exosome isolation strategies including ultracentrifugation, OptiPrep™ density-based separation, and immunoaffinity capture using anti-EpCAM-coated magnetic beads is performed by detecting exosome markers. Human colon cancer cell line LIM1863-derived exosomes based on the number of MS/MS spectra identified for exosome markers and proteins associated with their biogenesis, trafficking, and release; the researchers found IAC-Exos to be the most effective method to isolate exosomes. For example, Alix, TSG101, CD9, and CD81 were significantly higher (at least 2-fold) in anti-EpCAM-coated magnetic beads than the other two methods (Tauro et al., 2012). A comparative evaluation of ultra-centrifugation, polypeptide precipitation (ME Kit, NEP), and submicron size supermagnetic beads (SMB) with anti-CD9 confirmed SMB as a method of choice for plasma exosome enrichment; as the result of Western blot and FACS (fluorescence-activated cell sorter), the analysis verified multifold increase of exosome specific protein comparing to exosomes purified *via* other methods from plasma of the same volume. Moreover bead-based assays allow simple and rapid protocol in comparison to the plate-based ELISA (Zarovni et al., 2015). The magnetic bead-mediated selective adsorption strategy (MagExo), which tends to adsorb EVs on the surface of magnetic beads selectively, can separate EVs from plasma and cell culture media (CCM) with high purity, resulting in two times higher yield than EVs obtained by ultracentrifugation (Figure 1; Fang et al., 2021). However, in a comprehensive evaluation of differential centrifugation coupled with ultracentrifugation, epithelial cell adhesion molecule (EpCAM)-coupled microbead, and OptiPrepTM density gradient separation, the microbead shows inferior performance of purified exosomes. In Western blot analysis, less exosomal markers including HSP70, CD9, FLOT1, and CD63 are detected in exosomes purified by EpCAM-coupled microbeads. Microscopic analysis showed that the purified exosomes contain a large number of background proteins (Kalra et al., 2013).

## Discussion

In practical experiments and clinical studies, the most suitable method for exosome isolation is usually chosen based on the objective factors such as sample type, downstream experiments, study target, and rigid experimental conditions. Compared with the traditional methods, the method of extracting exosomes by magnetic beads can obtain a considerable number of high active exosomes. The magnetic bead-based method has better repeatability than the latex bead-based method, and increased capture efficiency and sensitivity compared to the plate surface-based method due to the larger surface area. However, the magnetic bead-based method has its own limitations. Considering that the magnetic bead-based method has not been standardized, it is recommended to conduct necessary identification, characterization, and functional experiments on exosomes in order to rule out the possibility of other impurities. Now neither the magnetic bead-based method nor other methods can completely isolate exosomes from other EV subsets. Also, several factors such as incubation time, temperature, level of surface markers expression, concentration of target vesicles state, characteristics of the antibody–antigen interaction, sample type, concentration and ratio of beads, and target molecules will have influence on the efficiency of the magnetic bead-based separation (Sioud, 2015). Magnetic beads only capture exosomes with target proteins on membrane surfaces selectively, so the yield of exosome is limited (Zhu et al., 2020). More targets coated on magnetic bead, stabler separation device, and combination of different approaches will be the future direction to boost the yield of exosome isolation. The advance of emerging strategies for labeling and tracking of exosomes will also promote the progress of the magnetic bead affinity method (Betzer et al., 2020; Thapa et al., 2019), and the technical improvements of exosome extracting, labeling, and tracking are expected to greatly facilitate exosome-based medical applications.

To sum up, the magnetic bead affinity method is an ideal method to enrich EVs including exosomes. However, for clinical application, ultracentrifugation and ultrafiltration are still the best alternative methods; we still have a long way to go before the magnetic bead affinity method being used in clinical diagnosis.
